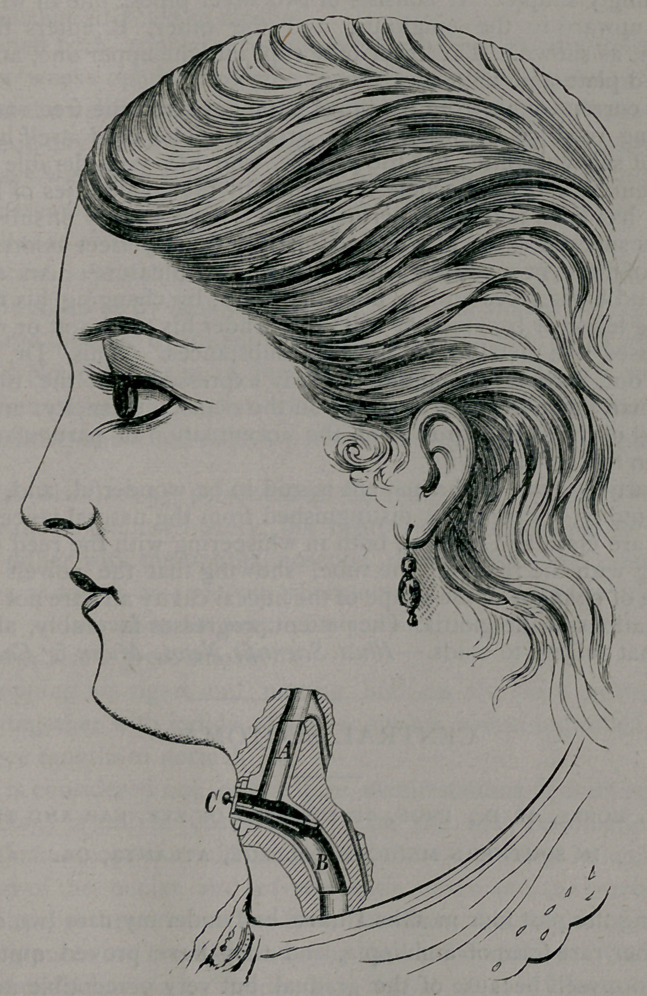# A Mechanical Larynx

**Published:** 1881-09-20

**Authors:** 


### A MECHANICAL LARYNX.

A remarkable cxample of how mechanical ingenuity is called in to supplement surgical skill is furnished by the successful extirpation of a larynx by Dr. Foulis, of Glasgow, and the substitution therefor of a metal contrivance, which supplies the place of the lost organ so perfectly that the patient is able to talk with as little difficulty as if the operation had not been performed. For the benefit of the reader not familiar with the functions of the larynx, we may recall the fact that the voice is produced therein by the vibration of the column of air passing through a narrow slit which forms the entrance to the trachea and lungs. The natural mechanism of the larynx is closely analogous to that of a reed instrument, in which a column of air, passing forcibly through a narrow slit bounded on one or both sides by a thin elastic plate of wood or metal, first causes the edge of the plate to vibrate with sufficient rapidity, and is thus itself thrown into sonorous vibration. In the larynx every variation between the two extremes of high and low notes is produced in similar manner by alterations in the width of the slit and the length and tension of its vibrating edges or vocal cords. When, therefore, a person is deprived of his larynx he becomes like an organ without pipes. The lungs, which correspond to the bellows, are there, and so is the articulating apparatus, which answers to the keys, but there is no means of producing sound.

Dr. Foulis' voice tube, which is represented in our engraving, is exceedingly simple. It consists of two silver pipes, one of which, A, passes upward to the epiglottis, and the other, B, enters the open trachea, as shown. The lower tube slips into the upper one, and holds the reed plate and button, C.

The current of air from the lungs impinges upon the free end of the vibrating reed, as shown by the arrows. The reed itself has been made of soft vulcanite; but the patient, who has considerable mechanical genius, and has become interested in the possibilities of his new larynx, has been making experiments on a large variety of substances, and has succeeded in providing himself with a perfect assortment of voices of different qualities, which he uses at pleasure. An alloy of silver and brass gives him a fine, rich tone; by changing his reed he can sing tenor or barytone at will, and render his tones soft or ringing, as he uses non-metallic or metallic substances. This, Dr. Foulis points out, proves the opinion already expressed, that the timbre of the human voice depends as much on the density, elasticity, and other qualities of the vocal cords as on the accentuation of particular sound waves in the buccal cavity.

The articulation of the patient is said to be wonderful, and, saving its monotony, it cannot be distinguished from the natural voice. The vowels are clear and distinct, both in whispering with the reed out and intoning with the reed in the tube, showing that the vowels are the product of changes in the shape of the buccal cavity and are not formed by alterations of the glottis. The patient progresses favorably, although somewhat subject to colds.—*Illust. Scientific News, Munn & Co., N. Y.*

## Figures and Tables

**Figure f1:**